# Emotions in simulation-based education: friends or foes of learning?

**DOI:** 10.1186/s41077-021-00198-6

**Published:** 2022-01-20

**Authors:** Vicki R. LeBlanc, Glenn D. Posner

**Affiliations:** 1 Department of Innovation in Medical Education, University of Ottawa, 850 Peter Morand Crescent, Room 102C, Ottawa, Ontario K1G 5Z3 Canada; 2 Department of Obstetrics and Gynecology, University of Ottawa, 501 Smyth Road, Ottawa, Ontario K1H 8L6 Canada; 3 University of Ottawa Skills and Simulation Centre, University of Ottawa and The Ottawa Hospital, 725 Parkdale Ave, Ottawa, Ontario K1Y 4M9 Canada

**Keywords:** Emotions, Learning, Simulation, Emotion regulation, Attention, Memory, Decision-making, Motivation

## Abstract

In simulation-based education, there is growing interest in the effects of emotions on learning from simulation sessions. The perception that emotions have an important impact on performance and learning is supported by the literature. Emotions are pervasive: at any given moment, individuals are in one emotional state or another. Emotions are also powerful: they guide ongoing cognitive processes in order to direct attention, memory and judgment towards addressing the stimulus that triggers the emotion. This occurs in a predictable way. The purpose of this paper is to present a narrative overview of the research on emotions, cognitive processes and learning, in order to inform the simulation community of the potential role of emotions during simulation-based education.

## Our beliefs as simulation educators

In simulation-based education, there is growing interest in the effects of emotions on learning during simulation sessions. Simulation educators firmly believe that active participation in a simulation scenario is more engaging than passively attending a lecture. Citing Russell’s circumplex model of emotions, many educators believe that emotional engagement and emotional realism in a scenario are important for buy-in, which in turn promotes learning [1]. The belief is that having activated learners, regardless of whether that activation is positive or negative, will lead to better learning. Some believe that there is a “sweet spot” of emotion for learning, where too little is not engaging enough, but too much is overwhelming [2].

Others argue that one particular emotional response—stress—helps with memory retention and therefore should translate into greater learning. The idea is that if information is encoded while in a stressed state, learners will be better able to consolidate this information in memory and to retrieve it later under similar circumstances [3]. In contrast, others argue that too much anxiety creates unsafe learning environments and that it impairs what is recalled and learned from these environments [4, 5].

Anecdotally, others advocate for evoking strong emotions at the simulation center so that trainees can practice functioning at that level, as a form of stress exposure. At a later date, when facing similar stress in real clinical situations, learners will be better prepared to deal with them.

Much of these beliefs are based on personal experiences as learners and teachers, but only variably based on evidence from research. The purpose of this paper is to present a narrative overview of the research on the interplay between emotions and the cognitive processes underpinning learning, in order to inform the community of the potential role of emotions during simulation-based education. Although simulation researchers have begun to explore the effects of emotions on learning and performance, the simulation literature is relatively sparse on this topic. As such, this review is primarily based on pertinent work from the domains of neuroscience and cognitive sciences, supplemented by findings from the field of simulation. For more thorough reviews of the domain of emotions, and how they relate to health professions education, readers are directed to recent reviews [6–9].

## What are emotions?

Emotions are complex processes with many components [10]: the conscious experience (e.g., feeling happy); facial, vocal, and postural expressions (e.g., smiling, upright posture); and physiological responses (e.g., increased heart rate and respiration rate), as well as motivation/behavioral intentions (e.g., strong desire to attend to a situation). Emotions are evoked in response to individuals’ appraisal of situations in relation to their goals, abilities, sense of control, and agency [11]. In turn, a given emotion’s motivational and physiological responses serve to organize behavior, cognition, and physiology in increasingly predictable ways, to rapidly address the situation [11]. Early models of emotions organized them around dimensions, such as valence (positive vs negative), arousal level (high vs low), motivational direction (avoidance vs approach), or combinations of these dimensions (circumplex model) [6]. According to these dimensional models, all emotions within a similar dimension (e.g., positive emotions, negative high arousal emotions) influence cognition and behavior in the same way [12, 13]. However, there is increasing support for discrete models of emotions, which postulate that different emotions have distinct effects on cognition because of their specific antecedents and physiology [14, 15, 16, 17]. For example, happiness signals satisfaction with one’s circumstances. It serves as a signal that one can down-regulate efforts related to a goal, in favor of savoring and celebrating the moment. This can lead to decreased motivation to immediately persevere towards one’s goals, as well as to more cursory processing of information from one’s surroundings. As for fear, it signals a situation that is potentially threatening to one’s goal. As such, it motivates caution and avoidance of harm, and its’ physiological response leads to heightened vigilance and preparation to flee. In this paper, because research on the effects of emotions has been based on both dimension and discrete models of emotions, we variably refer to emotions in their discrete (e.g., anger, anxiety) or dimensional (e.g., positive, negative) forms depending on the cited studies.

## What does the literature on emotions tell us?

The perception that emotions have an important impact on performance and learning is supported by the literature. Emotions are pervasive: at any given moment, individuals are in one emotional state or another. Emotions are also powerful: they guide ongoing cognitive processes by directing attention, memory, and judgment towards addressing the stimulus that triggers the emotion. This occurs in a predictable way, referred to as mood congruent processing [18]. As described in the sections that follow, we selectively pay attention to, remember, and interpret information that is congruent with our current emotional state, and our emotions influence our motivation and approaches to learning.

### Attention

One process that is essential for learning is attention. Because our capacity to attend is limited, we cannot process all of the stimuli that we encounter in our world. Attention processes are required for us to take notice of the more pertinent information in our environment, as well as to recognize objects. In turn, what we attend to has an important impact on what we extract from the world, how we experience the world around us, and what we remember from events [19]. This attentional selection is biased toward mood-congruent stimuli [19]*.* That is, we preferentially pay attention to information that is congruent with our current emotional state, particularly when experiencing negative emotions such as anxiety [20]. Emotional information is detected and identified faster, and we are more likely to interpret ambiguous information in line with our emotional state. For example, someone in an anxious state, who hears the word “beat,” is more likely to think of the action of striking someone than the rhythm of a song. If we extend this to the simulation environment, a learner who is anxious about being observed in a simulation session is more likely to pay attention to the observer’s reactions than to the details of the scenario. In contrast to negative emotions that are associated with a narrowing of attention onto stimuli that are congruent with that state, positive emotions can lead to greater distractibility [21].

### Memory

A second process that is essential for learning is memory. Emotions influence both what we remember *from* an event, as well as our ability to recall previous information *during* emotional situations. Memories from emotional events—particularly negative ones—tend to be more vivid, long lasting and detailed than memories from neutral events [22]. This has led to the belief that memory for emotional events is enhanced and more accurate. However, the impact of emotions is influenced by the relationship between the emotion and the various memory processes.

A significant amount of research has been conducted looking at the relationship between stress/anxiety and memory [23]. When stress is experienced immediately before or during encoding (e.g., initial storage of information into memory), memory will be enhanced for the information encountered. In contrast, if the stress occurs as little as 30 min before encoding new information, memory for that new information is subsequently impaired. Similarly, if stress is experienced when trying to retrieve previously learning information, or to update prior knowledge, then memory is impaired [23]. Therefore, there is some support for the notion that we remember more information from stressful events. During stressful events though, we are less able to retrieve information from memory [23].

An important caveat is that quantity does not equate to quality. Although emotions experienced at the time of learning new information can affect how much of that information we remember, there are some important biases in the details of those memories. After an emotional event, individuals are more likely to remember the elements that were more centrally related to the emotional trigger—in time, space and concept—than more peripheral information [24]. A powerful example of this is the “weapons focusing” observed during eyewitness testimonies [25]. Victims of crime are less able to recall the details of their assailant if there was a weapon present, yet they can describe the weapon in great detail. Because of the threat from the weapon, it captures their attention and is thus better remembered. This comes at the expense of details that are peripheral to the weapon, such as the appearance of the assailant. During a simulation session, this could manifest itself as the learner, who is anxious about being observed, remembering more information about the observer’s reactions at the cost of poorer memory of the details of the scenario itself.

In addition to the centralization of memory described above, strong emotions can also be associated with incorrect reconstruction of the past. Memory is not a true snapshot of a previous event. Rather, it is partly reconstructed based on our scripts, which are mental representations of what generally happens in a particular type of situation [25]. For example, if you are asked to remember and recount your experience of going for dinner at an upscale restaurant several months ago, your memory will be based both on the specific event that occurred, as well as on your script relating to dining at upscale restaurants: you are greeted by a host and shown to a table; the waitstaff will offer you water, hand you a menu, and then take your order after giving you time to look at the options. The bill will be presented at the end of the meal, you will pay at the table, and gratuities are generally expected. A significant amount of your recounting of your dinner experience will not be based on remembering all the specific elements that occurred. Rather, your memories are reconstructed because there was nothing unusual about the event. Therefore, you reconstruct your memory of the dinner based on your script.

Research shows that when people are exposed to a highly negative event, their memories tend to rely more heavily on this reconstructive process. When individuals were asked to recount what they experienced on September 11, 2001, when airplanes were flown into the World Trade Centre in New York City, 97% of respondents reported remembering exactly where they were, what they saw, and what they experienced. However, 73% of the respondents also recalled (incorrectly) that they saw the first plane striking the towers on the television [26]. Their memory of the event was strongly affected by their knowledge of what happened. This phenomenon has also been shown in the simulation setting with paramedics. Compared to when they completed a low stress scenario, paramedics were more likely to recall events that had not occurred after completing a high stress scenario [27]. Similar effects have been shown with happiness: People are also more likely to falsely report information based on their schemas or general knowledge when feeling happy [28].

Together, the results show that emotions do, in fact, have strong effects on our ability to recall information. Following an emotional event, particularly a stressful one, we recall more details related to the emotional trigger. However, due to attentional narrowing that occurs with emotions, those memories will have important biases. In summary, we might be able to remember more information from emotional situations, but those memories may be inaccurate and biased.

### Cognitive flexibility

Cognitive flexibility and the ability to form associations between two events or concepts (called associative learning) are also important components of learning that are affected by our emotional states. Positive emotions are associated with greater cognitive flexibility as well as openness to new information [28, 29]. These two processes are critical for learning and solving new problems. In contrast, negative emotions are associated with greater perseverance of inaccurate strategies (e.g., fixating) when trying to solve a problem and decreased ability to make associations between events (an essential component of learning).

Positive emotions are also linked with global processing: seeing “the forest for the trees” [28]. In contrast, people who are feeling sad are more likely to focus on the details, or local processing, rather than the big picture [29]. Global processing is linked with a greater ability to make associations between relevant learning events. This has implications for simulation sessions, in which educators aim to close performance gaps by correcting inaccurate learner frames. They strive to do so in a learner centered manner, by encouraging the learners to generate different ways of approaching similar problems and to generalize this to the real clinical setting. In these situations however, learners in negative emotional states may be less able to come up with different ways of doing things or to consider how the situation would generalize to the real world setting.

### Motivation and learning

Emotions can also have an impact on a learner’s motivation and efforts towards understanding educational materials, that is, their preparation, perseverance in the face of challenges, and strategies towards learning [9, 30]. Positive emotions, such as enjoying a task, can lead to greater interest and greater intrinsic motivation to engage in the task for its own sake [31, 32]. In contrast, negative emotions (e.g., boredom, anxiety, anger) can decrease interest and intrinsic motivation in a task. However, negative emotions can also increase extrinsic motivation; that is, motivation to engage in a task as a means to an end [33]. For example, the fear of performing badly in front of colleagues or of harming a patient may result in greater extrinsic motivation, thereby motivating learners to engage in behavior to enhance their learning. As such, both positive and negative emotions can enhance motivation to learn and thus subsequent performance [30]. These effects may be different for specific emotions. For example, Zhao [34] observed that fear did not have any significant impact on motivation to learn but had a direct negative effect on learning itself. In contrast, guilt and sadness were positively associated with motivation to learn but had no direct effects on learning from errors. Another study suggests that transient shame can lead to greater attention to feedback [35]. In contrast, deactivating positive emotions, such as relief, can have a detrimental effect on learning motivation and behaviors [36]. In a study of medical students’ learning with a virtual patient simulation program, relief was negatively associated with attention to feedback [35].

In the simulation environment then, the emotions experienced by the learners, as well as their attribution of the cause, can influence their motivation to learn from a session. It can also influence their resulting behavior related to learning. Learners who experience negative emotions because they feel like they did not know what to do may be motivated to engage more in the debriefing and to subsequently read more on the topic. In contrast, learners who experience negative emotions because they feel they were tricked by the educator may be less motivated to engage in the debriefing and are unlikely to seek to learn more on the topic after the session. In this case then, conceptual realism in simulation likely has an important impact on the motivational impact of the emotions experienced during the simulation sessions.

In summary, the literature from the neurosciences and cognitive sciences reveals that emotions play an important role in our thinking processes and our behaviors around learning. We are more likely to pay attention to information related to the cause of our emotions and to remember more of this information. However, this increased memory for emotional stimuli (particularly stressful ones) comes at a price. Following stressful situations, we are less likely to remember information that was peripheral to the emotion-causing event. As well, memory for emotional events is more likely to be biased based on our expectations and habits. Furthermore, emotions will influence our ability to make associations between events as well as our ability to demonstrate flexibility when solving problems. Finally, emotions will influence our motivation and behaviors related to learning activities.

### Emotions in health professions education

Although there has been limited attention placed on the role of emotions in learning in health professions education, early work shows similar effects as those observed in other fields. For example, during clinical reasoning, positive emotions have been linked with a more thorough diagnostic approach, decreased anchoring bias, and greater cognitive flexibility and creativity, as well as increased transfer of knowledge to new problems [37–39].

There is also preliminary work looking at the effects of emotions on learning. In one study, DeMaria and colleagues observed increased performance on a simulated Mega Code scenario 6 months after a stressful simulation-based education session [40]. In contrast, Fraser and colleagues observed decreased performance on a toxin-ingestion OSCE 3 months following a stressful simulation-based education session [41]. More conflicting results come from McConnell and colleagues, who observed decreased learning following both a positive and a negative mood induction (being asked to recall either a positive or a negative event from their past), compared to a neutral condition [42].

## Implications for simulation-based education

As simulation educators, our primary goal is to ensure that our learners attend to and learn from the educationally salient parts of our teaching session. Thus, the effects of emotions on learning and problem solving present the field with an important quandary. On the one hand, we are tempted to explicitly manipulate our simulation scenarios to activate emotions, in the hopes that we can link these emotions with the critical aspects to be learned, and thus enhance our learners’ memories. On the other hand, the conflicting data from the literature and the identified biases can lead others to seek to reduce the emotional elements of simulations, with concerns that they create more havoc than anything else. Both tendencies come with important risks. If we explicitly attempt to manipulate our learners’ emotions, this could backfire on us due to their effects on attention, memory, cognitive flexibility, and motivation. In the converse, it would be a fallacy to push emotions aside in our attempts to focus on the more “rational” cognitive elements of learning. Emotions are powerful and pervasive. Attempts to suppress or avoid emotions are maladaptive coping mechanisms that have been linked with greater likelihood of developing post-traumatic stress symptoms as well as poorer physical health [43, 44].

While further research is needed to better understand the interplay between emotions and our cognitive processes, both in the laboratory and in applied settings, there are some actions that educators can take to address emotions during simulation activities:

1. Simulation educators should be thoughtful and deliberate regarding the emotions triggered in their activities. In some situations, educators may want to trigger an emotional reaction in their learners, to prepare them for the emotional states likely to be encountered in clinical settings. In these cases, this should be an explicit objective of a simulation session. Additionally, exposure alone is not sufficient for individuals to learn adaptive coping strategies. Exposure should be supported with explicit education and practice around adaptive coping strategies. Educational strategies targeting emotional regulation are discussed in more details later in this paper. In these situations, it is important to keep in mind that in mood manipulation experiments, individuals do not always experience the target emotions [45]. As such, educators should expect that some learners in the group may respond differently than expected, and be prepared to adjust accordingly.
2. In simulation sessions where emotional regulation is not an explicit learning objective, educators should be cognizant of the various ways that a simulation session can create unexpected emotional reactions in learners, as well as how these emotions could bias the learning that occurs from this session. Conditions that can inadvertently create emotional reactions that pull the learners’ attention away from the learning objectives include the absence of psychological safety (discussed in more details below), peripheral challenges that create extraneous cognitive load [46], levels of challenge that are too low or too high for the learners’ level of knowledge and skills [47], learners feeling tricked [48], the perception of being observed or evaluated, competing demands on the learners’ time spent in a simulation session, fear of failure, and exposure to discourteous behavior [49, 50], as well as emotional contagion [51] from the facilitator or fellow learners. As well, the delivery of feedback itself can evoke unexpected emotional reactions in learners [52–54]. In turn, this can affect how the learner receives and processes that feedback [55]. This is relevant for not only debriefing sessions following mannequin-based sessions, but also in a skills-based session where learners are acquiring procedural skills.
3. Despite the aforementioned advice to limit extraneous elements that could evoke emotions that orient the learners’ cognitive processes away from the learning objectives, we are intimately aware that emotions are a constituent part of many simulation sessions. The very strength of simulation is that it places learners in situations that recreate the demands and conditions of the real world—in ways that challenge their knowledge, skills and attitudes—for the sake of new learning. These are the very conditions that can provoke emotions. As such:
  - When emotions are likely to be triggered during a simulation scenario, care should be taken to ensure that the emotions are linked (in time, space and concept) with the to-be-learned information and that the emotional realism of the scenarios is high. For example, rather than triggering anxiety through irrelevant peripheral distractors (i.e., the program director is observing today), anxiety could be triggered by a rare disease presentation (i.e., eclampsia), in time-pressured situations, with high stakes (abnormal fetal heart rate tracing).
  - Ensure psychological safety during the simulation activities. During formative learning sessions, any performative elements should be removed, and a climate that minimizes fear of mistakes should be sought. This includes not only following principles of pre-briefing (i.e., learning contract, trust, respect) [56, 57], but also fostering a broader institutional culture of learning.
  - Educators should be watchful for strong emotions in learners and seek to diffuse those emotions that could negatively affect learning. In addition to self-reports, individuals manifest emotions through facial expressions, body language and posture, as well as speech (e.g., intonation, pitch, rate, loudness) [10, 58]. These become particularly interpretable as emotions become more intense [59]. Although recent developments in technology have made physiological monitoring more accessible and attractive to educators, their usefulness for detecting emotions other than stress are limited [60]. If strong emotions are present and felt to be detrimental to learning, one strategy that can minimize the effects of emotions is to draw attention to them and label them [61]. Therefore, although there is currently little formal evidence to support debriefing approaches that directly target emotions (such as the reactions phase of the PEARLS framework [62]), there is strong theoretical support for such approaches.
  - In situations where labeling emotions are insufficient to diffuse them, educators can engage in extrinsic (or interpersonal) emotional regulation [63]. Extrinsic emotional regulation consists of actions performed with the goal of influencing another person’s emotional state, to decrease or increase either negative or positive emotions. Common examples include situation modification (changing a situation by removing some or all of the emotion-provoking elements), attention deployment (selecting which aspects of a situation to focus on by distracting attention away from the elements that are harmful to the learner’s goals, concerns or wellbeing), cognitive change (selecting which of many possible meanings to attach to a situation) and the often less effective modulation of the emotional response (actions to suppress the emotional response by influencing physiological, experiential or behavioral responding—e.g., directing someone to “take deep breaths” or to “calm down”) [64, 65]. For example, if an educator recognizes that a learner is disengaged due to shame at being unable to manage a particular situation or skill, she could help the learner normalize the experience and reframe the situation as an opportunity for curiosity and growth, and thus motivate the learner towards seeking strategies to master the challenge.
  - Following simulation scenarios that are likely to trigger strong emotions, educators should consider the use of educations adjuncts to reinforce the key learning information. For example, consider having written information of key points that learners can take away with them (e.g., guidelines, decision charts related to the scenario content, cheat sheets). As well, more experienced debriefers could supplement their sessions by documenting key learning points on a white board and encouraging learners to copy or photograph these points for further reflection or review. These will help reinforce the desired learning. This also reinforces the importance of strategies such as reviewing, at the end of a debriefing session, the main learning points and asking learners to highlight their key learning from the session.

### Preparing for emotional situations

In situations where the explicit learning objectives include exposure to emotion-inducing situations (e.g., stress exposure), adaptive emotional regulation strategies should be explicitly taught with opportunity to practice. Emotional regulation consists of individuals consciously modifying their emotional responses to a situation [66]. Currently, the onus is most often placed on individuals to develop their own coping skills. When left to their own devices, individuals often develop maladaptive coping skills (e.g., suppression) that are associated with poorer performance and poorer emotional control, as well as increased likelihood of long term mental and physical sequelae [43, 67]. As such, we need to consider formally preparing individuals to use adaptive emotion regulation strategies in emotional situations [66].

Simulation can be used to teach learners how to recognize their emotional state (e.g., mindfulness) and engage in adaptive emotional regulation (e.g., reappraisal) in situations where their emotions could present a problem. In these types of sessions, the educator’s role is to help learners recognize their emotional state, assess whether that emotional state is beneficial or harmful to the situation, and learn how to regulate their emotions in those situations where the emotions are detrimental to the situation. Adaptive emotional regulation strategies include normalizing the reaction, mindfulness, deliberate relaxation, modifying the situation, and reappraisal of the situation [66]. Depending on the circumstance, distraction may be beneficial or detrimental. Less effecting strategies are those related to emotional suppression [43, 67]. One example of emotional regulation training used to prepare for high-anxiety provoking situations is stress inoculation training [68, 69], where individuals learn to recognize their early signs of stress/anxiety, then practice relaxation (e.g., breathing techniques) and cognitive reappraisal skills (e.g., reframing the problem) in low stress environments before applying them in controlled situations meant to systematically increase the level of stress. Simulation-based sessions are particularly well suited for stress inoculation and other forms of emotional regulation training.

## Conclusion

In summary, simulation-based education can be rife with emotional situations. The emotional reactions experienced by individuals during simulation can have significant effects on what they attend to, what they remember from these events, their judgments and problem-solving approaches, as well as their motivation to engage in learning behaviors. These emotions are neither good nor bad, they simply *are*. In some cases, emotions will enhance how we interact with the world around us, while in others they will impair it. To better support learners, simulation educators must gain a greater understanding of the critical role of emotions in how individuals interact with their environments. Doing so will allow for a greater ability to support learners with teaching adjuncts following highly emotional situations, to apply effective extrinsic emotional regulation strategies when needed, as well as preparing learners to recognize and adaptively regulate their emotions when caring for patients in uncertain and sometimes challenging situations.

## Data Availability

Not applicable
